# Phantom controllers: Misspecified models create the false appearance of adaptive control during value-based choice

**DOI:** 10.1101/2023.01.18.524640

**Published:** 2025-04-14

**Authors:** H. Ritz, R. Frömer, A. Shenhav

**Affiliations:** 1 Cognitive, Linguistic, and Psychological Sciences, Brown University; 2 Carney Institute for Brain Sciences, Brown University; 3 Princeton Neuroscience Institute, Princeton University; 4 School of Psychology, University of Birmingham; 5 Centre for Human Brain Health, University of Birmingham; 6 Department of Psychology, University of California, Berkeley; 7 Helen Wills Neuroscience Institute, University of California, Berkeley

## Abstract

Decision scientists have grown increasingly interested in how people adaptively control their decision making. Researchers have demonstrated that parameters governing the accumulation of evidence towards a choice, such as the decision threshold, are shaped by information available prior to or in parallel with one’s evaluation of an option set (e.g., recent outcomes or choice conflict). A recent account has taken a bold leap forward in this approach, suggesting that adjustments in decision parameters can be motivated by the value of the options under consideration. This motivated control account predicts that when faced with difficult choices (similarly valued options) under time pressure, people will adaptively lower their decision threshold to ensure that they make a choice in time. This account was supported by drift diffusion modeling of a deadlined choice task, demonstrating that decision thresholds decrease for difficult relative to easy choices. Here, we reanalyze the data from this experiment, and show that evidence for this novel account does not hold up to further scrutiny. Using a more systematic and comprehensive modeling approach, we show that this previously observed threshold adjustment disappears (or even reverses) under a more complete model of the data. Importantly, we further show how this and other apparent evidence for motivated control arises as an artifact of model (mis)specification, where one model’s putatively controlled decision process (e.g., value-driven threshold adjustments) can mimic another model’s stimulus-driven decision processes (e.g., accumulator competition or collapsing bounds). Collectively, this work reveals crucial insights and constraints in the pursuit of understanding how control guides decision-making, and when it doesn’t.

Recent years have seen a growing interest in the intersections between value-based decision-making and cognitive control^1^, providing researchers with fresh new insights into how people decide how to decide. These lines of research have demonstrated that decision-making is not a purely stimulus-driven process – one in which evidence accumulates based entirely on the values of one’s options – but, rather, that evidence accumulation is subject to adaptive and online control. For instance, a substantial body of work has now demonstrated that people can adjust their decision threshold (how certain they must be to make a choice) by leveraging metacognitive information about this decision process. Decision-makers increase this threshold when they have conflicting evidence in support of multiple options (i.e., to buy additional time to resolve conflict and avoid mistakes^2^), and they lower (collapse) this threshold over the course of a decision, particularly when there is an impending deadline (i.e. reflecting the increasing urgency to ensure some response is made in time^3–6^ ). An influential new theory has proposed a bold extension on these prior accounts: that people may adjust their decision threshold not only based on metacognitive information about the task and decision process, but also based on direct access to the values of the options themselves.

Drawing on prior research on motivated control^7,8^, Vassena and colleagues recently proposed that when people make deadlined choices, they make two parallel decisions based on the values of their options^9^. One of these decisions involves accumulating evidence for these options until reaching a threshold to select the best one (i.e., classical evidence accumulation). The other decision involves determining whether the values of their options merit lowering that decision threshold. To the extent that the value of one option is clearly higher than the other, their model predicts that decision-makers should maintain their typical threshold. To the extent these option values are similar, however, their model suggests that a person should lower their decision threshold and make a more immediate decision, otherwise risking the possibility of failing to choose any option. For the same reason, a person should especially lower their thresholds (“invigorate” their response) when choosing between options of similarly high (vs. similarly low) value, to avoid an even larger loss if failing to make a choice before the deadline^10^.

Vassena and colleagues’ motivated control account shares properties with metacognitive accounts above, but it differs in two critical respects. First, it assumes that people are adjusting their threshold based on the specific values of their options rather than based on the environmental statistics (i.e., agnostic to the properties of each specific choice) or based on metacognitive properties of the choice (e.g., their uncertainty in their option values or in the choice process as a whole, rather than specific option values). Second, and critically, it makes the *opposite* prediction from existing conflict-based metacognitive accounts, in that it predicts that difficult choices should entail *lower* rather than higher thresholds (compare ^2,11,12^).

To test this novel account, the authors had participants perform a value-based decision-making task, with each trial requiring them to select between two options within a short deadline. The authors analyzed choice behavior from this task using a drift diffusion model (DDM;^13,14^) and found that, as predicted, participants set lower decision thresholds when option values were more similar to one another (difficult choices) than when their options were more dissimilar (easy choices). Additional evidence supported the further prediction that participants lowered their thresholds for high-value relative to low-value option sets^9^. Collectively, these findings were taken as support for the proposal that people adapt their decision thresholds as a function of the expected rewards that the person is actively selecting between. Having behaviorally validated this motivated control account, the authors go on to present it as an alternative to their preexisting account of neural data collected during the choice task (the PRO model;^15^). They show that the motivated control account, despite seeming to offer a good account of behavior, is unable to explain neural activity during this task (in contrast to the PRO model).

Here, we revisit behavioral evidence for this novel motivated control account. We formally model the proposed account – that decision thresholds are adjusted based on the relative value of one’s options – and show that this model poorly accounts for participants’ behavior and neglects significant drivers of choice. Instead, we show that previous evidence for motivated control appeared artifactually due to multiple forms of model mimicry. First, we find that decision difficulty mimicked the influence of *overall* choice value (i.e., the average value of one’s options) on choice behavior. Second, we found that joint effects across drift rate and threshold mimicked a time-varying collapse in participant’s decision threshold. Finally, we found that a drift diffusion model with threshold control can mimic a control-free decision process using competing accumulators. Our findings undercut previous support for this novel motivated threshold account, and more broadly highlight significant risks for model mimicry that can occur when studying value-based decisions through an evidence accumulation framework.

## Results

**Figure 1.**
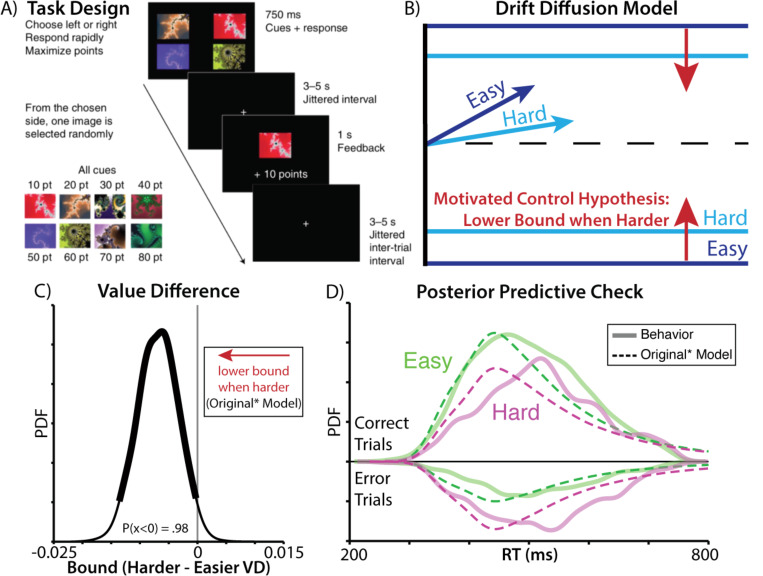
Predictions of the motivated control account are confirmed when specifying the drift diffusion model as in the original study. **A)** In this experiment, participants chose between two bundles of fractals worth a known amount of money. Time pressure was introduced by requiring that choices be made within a 750ms deadline, otherwise forfeiting any reward for that trial. **B)** The motivated control account predicts that harder choices (those involving similarly valued bundles; dark blue) should be associated with lower rates of evidence accumulation (as predicted by other accounts) and, critically, that these choices should also be associated with lower decision thresholds, relative to easy choices (light blue). **C)** Consistent with this account, when fitting drift diffusion models to these data using similar specifications as in the original paper, we find that harder choices are reliably associated with lower thresholds (97.5^th^ percentile of posterior density was less than 0). **D)** When comparing RT distributions predicted by this model (dashed lines) to the actual data (solid lines), we find that the predicted distributions are substantially more skewed than the actual ones, both for easy (green) and hard (purple) choices. See also Supplementary Figure 1. Panel A adapted from (Vassena et al., 2020).

Vassena and colleagues (2020) had participants perform a deadlined value-based decision-making task for monetary reward (Figure 1A). On each trial, participants had a limited time window (750ms) to choose between two bundles. Each bundle contained a pair of fractals, and participants had learned the fractal values during training. For instance, the left bundle could have fractals worth 70 and 90 points, and the right bundle could have fractals worth 30 and 40 points. After they chose their preferred bundle, participants received a monetary bonus based on the point value of a random fractal from the bundle. Participants were therefore incentivized to choose the more valuable pair within the time allotted, and it was better for them to make a choice than miss the deadline (i.e., options were entirely in the domain of gains).

Vassena and colleagues argued that under these conditions it would be most beneficial for the participant to lower their response threshold when their choice is more difficult (i.e., when the bundle values are most similar to one another), because otherwise they risk failing to respond before the deadline. In other words, participants should be motivated to exert control to reduce their response threshold (“invigorate” their response) when they discover that their option values are similar to one another. In a later elaboration of their account, they unpack that this motivation should be even stronger the more valuable their options are (i.e., the higher the average value across all fractals on the screen) given that these comprise the opportunity cost of failing to respond by the deadline^9^.

### Models with motivated control (value-based threshold) leave room for improvement

To support their hypothesis, the authors fit a hierarchical drift diffusion-model (HDDM) to participants’ accuracy and reaction times. They fit separate drift rate, threshold, and starting point parameters for easy and difficult trials, defined based on the absolute difference in bundle value (weighting the fractals equally; Figure 1B). When choices were more difficult, they found that drift rates were lower (consistent with weaker relative evidence for one option vs another^2^). Critically, consistent with their predictions, they *also* found that participants’ thresholds were lower during more difficult trials (percent of posterior density below zero: P(x < 0) = 0.98). Moreover, in support of the prediction that this threshold-lowering should scale with the overall value of one’s options, they went on to show that thresholds were also lower for choices with high relative to low overall value^10^ (controlling for difficulty).

Using the same HDDM model specifications provided by the original authors (‘Original Model’), we replicated all the findings reported above, most notably that drift rate and threshold vary with value difference in the predicted direction (Supplementary Figure 1A). However, these analyses also suggested that there was significant room for improvement in the model’s ability to capture choice behavior on this task: model-generated choices and response times, as originally specified, failed to provide a qualitative match to the empirically observed choice and RT distributions (Supplementary Figure 1B). For instance, their best-fit model predicted RT distributions that were substantially more skewed than the actual data.

As initial steps towards addressing these concerns of model fit, we generated a revised model that encompassed the spirit of the author’s original predictions, but included modest adjustments to better account for participants’ choice behavior (‘Original* Model’). First, to account for potential response biases (e.g., favoring the left or right option), we coded choices in terms of which side was chosen (i.e., ‘response-coded’ rather than ‘accuracy-coded’) and fit subject-specific starting points. Starting point now determined left/right biases, and not a bias towards the correct/incorrect choice on the basis on difficulty before any evidence has accumulated. Since choices were response-coded, we included the most difficult trials, which were removed in the original analysis. Second, we included an outlier term to account for attentional lapses. Third, we fit difficulty as a continuous variable, rather than a discrete variable, as the psychological construct is usually thought of as continuous and median splits (as used in the Original model) can reduce statistical power.

These model adjustments collectively led to quantitative improvements in model fit (change in DIC between Original and Original* model = 569), without altering any of the key observations: with this elaborated model, we still found that thresholds were significantly lower for difficult relative to easy choices (Figure 1C). However, when simulating distributions of choices and response times from this model, we found that these distributions still provided a poor qualitative match to empirically observed behavior (Figure 1D), predicting a much more heavily skewed RT distribution. This mismatch between model and data motivated us to consider additional revisions that would better account for core elements of the task and findings.

### Evidence for motivated control is eliminated when accounting for core elements of choice

Based on the design of the choice task and the patterns of behavior that were reported by the original authors, we considered three modifications to the elaborated model above, and tested each one in turn.

First, the models above assume that participants weighted all four fractals equally when choosing between the two bundles. As a consequence, value difference (and thus choice difficulty) is determined by comparing the average value of each bundle. However, this assumption is contradicted by the authors’ regression analyses showing that participants overweight the higher-value fractal within each bundle relative to the lower-value one (placing five times as much weight in the former than the latter)^9^. We therefore tested whether separately modeling the relative and overall influence of the higher-valued and lower-valued options in each pair improved model fit.

Second, the models above only consider how parameters of the DDM will be influenced by the *difference* between two option values. However, follow-up analyses by Vassena and colleagues^10^ report that these parameters also differ by *overall* set value (sum of left and right option values), consistent with a broader array of value-based decision-making research^16–19^. While the authors had originally designed their bundles to orthogonalize overall value and value difference, this was done under the assumption that participants weighed the fractals in each bundle equally. We reevaluated this assumption based on the unequal weights revealed in the analyses above, finding that these re-weighted estimates of overall value and value difference were now correlated (Supplementary Figure 2). We therefore tested whether DDM fits would be improved by including overall value as a predictor of drift and threshold in our model (along with value difference).

**Figure 2.**
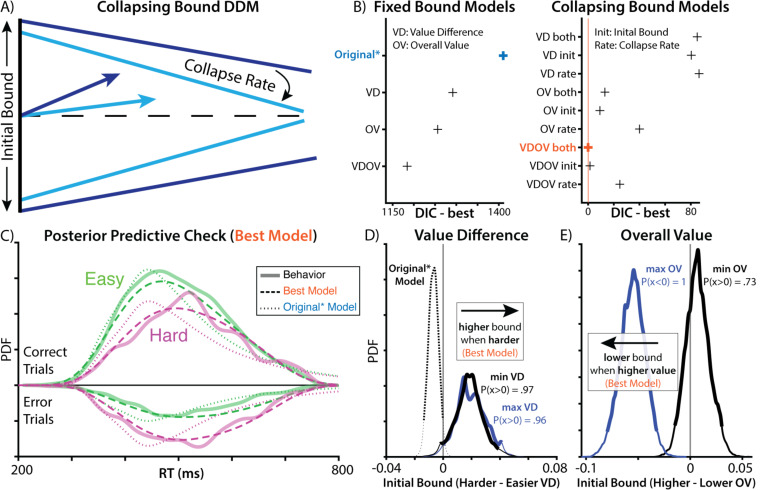
Elaborated models improve fit and contradict original findings. **A)** To account for the response deadline, we fit a DDM with a threshold that linearly decreases (collapses) over time rather than remaining fixed over an entire choice (as in the previous models). **B)** Complexity-penalized model comparisons show that incorporating this collapsing bound produces substantial improvement in model fit. Model fit further improves when allowing this collapsing bound to be further modulated by both value difference and overall (summed) value of the options, as well as when allowing for separate weights on the higher- and lower-valued fractals within each pair. **C)** Simulated behavior from the best-fitting model (dashed line) provides a substantially better qualitative fit to participant data (solid line) than the Original* model (dotted line; cf. Figure 1D). **D)** Under this best-fit model, we no longer see difficulty associated with decreases in threshold, but instead a trend in the reverse direction (compare Figure 1C). **E)** This model also shows that higher overall value is associated with lower thresholds, consistent with a separate prediction of the motivated control account.

Third, the models above assume that participants maintained a fixed response threshold throughout the choice period. Past work suggests that decisions under time pressure are instead often better characterized by a response threshold that decreases (collapses) over time, requiring less evidence over time but guaranteeing that a response is made before one’s deadline^4^ (Figure 2A). A collapsing threshold model differs from the motivated control account above in that it is agnostic to the value of one’s options on a given trial, instead assuming that thresholds collapse at the same rate on every trial (e.g., due to the deadline). In other words, a collapsing threshold, in and of itself, does not necessitate motivated control. We tested whether a collapsing threshold improved model fit relative to a fixed threshold model.

We found that models incorporating these modifications fit participants’ data far better than the Original* model (Figure 2B). A model allowing weights to differ between max and min values fit better than the original equally weighted model (ΔDIC = 119). Models with collapsing bounds substantially outperformed models with static bounds (Value difference [VD] model: ΔDIC = 1206, Overall value [OV] model: ΔDIC = 1243, model with both [VDOV]: ΔDIC = 1184). Critically, unlike models with static bounds, models with collapsing bounds were able to accurately capture the skew of participants’ reaction time distributions (compare dotted and dashed lines in Figure 2C). Both in models with static bounds and collapsing bounds, models that included an influence of overall value on threshold fit better than models in which overall value was excluded (static-bound VDOV vs. VD: ΔDIC = 107; collapsing-bound VDOV vs. VD: ΔDIC = 85).

We validated our model selection procedure using model recovery. We generated synthetic datasets from three models (Original*, VD static bound, VD collapsing bound) by sampling from participants posterior parameter distributions, and then we fit each model to each generated dataset. We found that our model comparison procedure was biased towards collapsing bound models, with better DIC even when the data were generated by a static bound model (Supplementary Figure 3A). However, these biases were an order of magnitude smaller than the empirical DIC differences from fitting these models to participants (Supplementary Figure 3B). Given how unlikely our empirical DIC differences were under the null distribution generated by our model recovery, as well as the striking differences in posterior predictive checks (Figure 2C), these findings continue to support the collapsing bound mechanism. Speculatively, our use of participants’ parameters may have found a region of the parameter space where there was particularly high mimicry between static and collapsing bound models. Another alternative that has been proposed to the collapsing bound mechanism is across-trial variability in drift rate^20^. However, we found that models with across-trial variability, but not collapsing bounds, provided a poor account of the RT distribution (Supplementary Figure 4A).

Tajima et al 2016 We validated the identifiability of these parameter estimates using parameter recovery in our best-fitting model: simulating datasets with ground-truth parameters, re-fitting the model to these datasets, and then measuring the similarity between ground-truth parameters and estimated parameters. We found that we had good parameter recovery (Supplementary Figure 5), improving our confidence in these parameter estimates.

Importantly, to the extent difficulty was associated with threshold adjustment, it was now in the exact opposite direction from what Vassena et al. described. Harder choices were now associated with *higher* rather than lower thresholds (Figure 2D), in contradiction with the predictions of their motivated control account. Instead of difficulty, the strongest influence on threshold was the overall value of the choice set (Figure 2E; see Supplementary Figure 5 for all group-level parameters), more consistent with previous work on value-based decision making ^16–19^.

### Evidence of motivated control can emerge as an artifact of model mimicry

Our findings so far undercut one major prediction of the motivated control account, which is that people use information about the similarity in their option values to adjust their threshold downward (i.e., to invigorate responses when choices are difficult). However, these models retain clear evidence for a revised account^9,21^ in which people use information about the overall option value to lower their threshold (Figure 2E). This leaves open the possibility that participants are engaging in a form of motivated threshold-adjustment, but one that is more sensitive to overall value than choice difficulty. However, previous research points to an explanation of these findings without invoking any form of motivated control.

**Figure 3.**
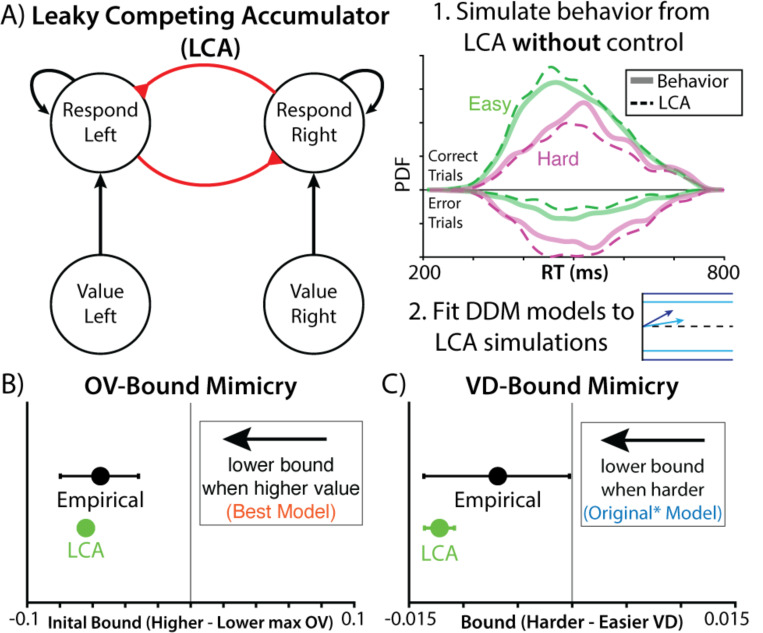
A control-free model can account for choice behavior while mimicking a motivated controller. **A)** To test whether stimulus-driven processes were sufficient to account for apparent evidence of motivated control, we simulated choice behavior from a leaky competing accumulator (LCA) that lacked a threshold control mechanism. We then fit these synthetic data to DDMs that assume that threshold is modulated by value as predicted by the motivated control account. **B)** Fitting these data to our best-fitting DDM (cf. Figure 2E), we see that the control-free LCA also produces artifactual evidence of overall-value-dependent decreases in threshold. **C)** Fitting these data to the Original* model (cf. Figure 1C), we see that our control-free LCA produces artifactual evidence of choice-difficulty-dependent decreases in threshold.

Prior work has shown similar forms of overall value-related speeding as observed in this study, showing that this behavior can naturally emerge from a stimulus-driven evidence accumulation process. In the leaky competing accumulator (LCA^22^; Figure 3A), where evidence accumulates for each response in parallel, more valuable options naturally produce stronger activations of potential responses, thus resulting in earlier threshold-crossing (see also ^16,17,19^). This is the case because unlike in the DDM, where inhibition leads to full cancellation and accumulation of the signed difference in evidence regardless of the overall value of options, in the LCA evidence for each option is accumulated in parallel with the accumulators mutually inhibiting each other proportional to their respective activations (see also ^23,24^). As a consequence, across all accumulators, activation raises faster for higher value options, and due to the non-linear properties of the mutual inhibition, whereby higher activation leads to stronger inhibition, activity also diverges faster, both resulting in faster response times for higher value option sets. Critically, this dynamic occurs *without* any additional control process to adjust decision thresholds. It is therefore possible that the apparent value-related control of threshold in the DDM could be mimicking an alternative decision architecture.

To test this, we simulated choice behavior in this experiment using an LCA that was coarsely tuned to mimic participants’ accuracy and reaction time (See ‘LCA Simulations’ in Methods). Like the DDMs described above, evidence accumulation in this model was determined by the values of the individual options, with the higher value options within a bundle taking on four times the weight of the lower value options, similar to what was empirically observed. The LCA makes a choice when one of its accumulators crosses a collapsing decision threshold. Critically, unlike the previous DDM-based models, value was not otherwise able to influence the decision threshold, having the same initial bound and rate of collapse across all trials.

Choices simulated from our LCA exhibited all the key patterns observed in the original data, including faster and more accurate choices when there were larger value differences between the options (Figure 2A), and faster responses with higher overall set values. The fact that this “control-free” model reproduces behavior on this task indicates that stimulus-driven dynamics are sufficient to account for such behavior without invoking motivated control. It also suggests that the threshold-related adjustments that were evident in our DDM analyses may have been illusory. To test this, we took choice behavior simulated by our LCA and fit it with our DDM models (Figure 2A). Remarkably, we once again find evidence that threshold significantly decreased with overall value, despite the *absence* of such a connection in the model that generated those data (Figure 3B). Thus, overall value-related threshold adjustments (within a DDM framework) artifactually mimicked the true effect of overall value driving choice in a stimulus-driven fashion (within an LCA framework).

We suspected that another form of model mimicry may have accounted for the other piece of evidence for motivated control in these data – the effect of value difference on decision threshold (lower thresholds for harder choices). Specifically, given that higher vs. lower value differences are associated with higher vs. lower rates of accumulation, respectively (under any of the models we’ve described), these decisions will reach a collapsing threshold when it is at a high vs. low point in its collapse. Thus, even though that collapsing threshold is only driven by time within a trial (i.e., it has no access to information about option value), the threshold level at which a decision is reached differs across the two cases. If a model omits this collapsing mechanism and instead assumes that thresholds are fixed within a trial – as was the case for Vassena et al.’s models ^9,10^ – then it may appear as though trials with lower value differences also have lower fixed thresholds. Moreover, confounding between overall value and value difference under unequal weighting (see Supplementary Figure 2) only exacerbates the potential for an artifactual influence of value difference. To test this, we again fit behavior generated by our LCA (which had a collapsing threshold) to the DDM that was consistent with the original authors’ hypothesis (which had a fixed threshold). We find that this analysis mistakenly identifies a positive relationship between value difference and DDM threshold, despite there being no such connection in the generative model (Figure 3C). Thus, a model that lacks motivated control of decision threshold can not only account for behavior on this task, but can generate behavior that can be confused for motivated control.

## Discussion

Decision scientists have grown increasingly interested in how we control our decision-making^1,25,26^. Building off of research into adaptive control mechanisms in other forms of cognitive tasks^27–29^, studies have shown that such decision parameters can be shaped by variables such as temporal urgency^4^ and choice conflict ^2,11,12^. Vassena and colleagues sought to break new ground in this area by proposing a new form of value-driven adaptive control that leverages information available during a choice (option values) to guide adaptations to that choice (threshold adjustments). They provided critical model-based evidence for this account by demonstrating that difficult speeded decisions were associated with lower decision thresholds than easy ones, consistent with adaptive reductions in threshold to avoid the possibility of failing to choose either option. We reexamined their findings with a more comprehensive modeling approach, and showed that this apparent value difference-related threshold decrease arose artifactually from misspecifications in their choice model. When all features of these speeded decisions were properly accounted for, hard decisions were if anything associated with higher rather than lower thresholds. We further showed that this and other value-driven threshold adjustment predicted by this account (e.g., lower thresholds for higher-valued sets) could collectively be accounted for by bottom-up decision processes expressed in a variant on the original modeling framework (LCA rather than DDM). Our findings thus provide alternative explanations for evidence that has been brought to bear on this novel account, while also highlighting important opportunities and pitfalls for future research into related mechanisms of adaptive control. We begin by addressing the latter before returning to the former.

**Figure 4.**
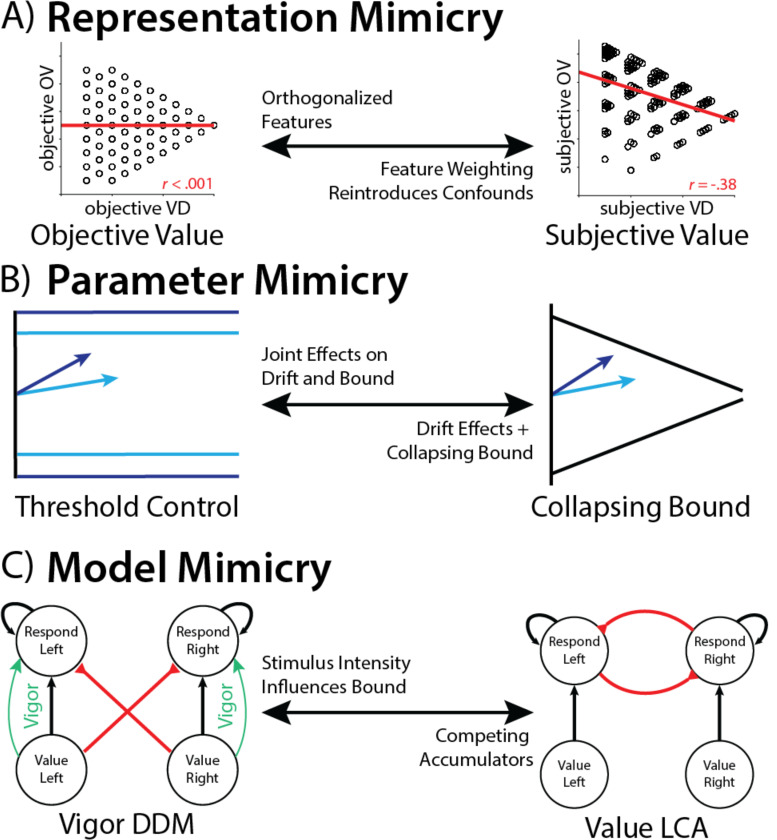
Multiple sources of mimicry in modeling value-based choice. **A)** *Representation mimicry* can occur if participants are using different information than the model assumes they use. In this experiment, participants’ strategy over-weighting of high-value fractals relative to low-value fractals hid the influence of overall value on decision-making when this weighting strategy was unaccounted for. **B)** *Parameter mimicry* can occur when different parameterizations can capture similar behaviors. In this experiment, the joint effects of drift rate and threshold mimicked the influence of a collapsing bound^21^. **C)** *Model mimicry* can occur when different model classes can capture the same behavior. In this case, the influence of overall value on decision threshold could be parsimoniously accounted for by intrinsic dynamics of an independent accumulator model such as an LCA (see also ^33^).

Our work highlights potential pitfalls for research into the computational basis of value-based decision-making. We show that even for a relatively simple choice involving two pairs of learned values, there were at least three major modeling decisions that substantively impacted the conclusions that were ultimately drawn (Figure 4). First, we showed that model fits were improved by allowing the values in each option pair to have separate weights in the decision rather than averaging these uniformly (Figure 4A). These differential weights were evidenced in behavior, and are consistent with previous work suggesting differential attentional priority under constrained time and attentional resources^30^. Failing to account for these asymmetric weights created the false appearance that value difference and overall value were orthogonalized across choices (as originally intended), whereas accounting for these correlations reveals that these variables were in fact correlated. Thus, results previously attributed to value difference (e.g., its predicted influence on threshold) were at least in part contaminated by effects of overall value. Second, we showed that model fits were improved by allowing thresholds to collapse over time within a trial (agnostic to information about the options themselves; Figure 4B). This is consistent with a wide array of previous findings that implicate such dynamic time-based threshold adjustments^3,4,6^, particularly in the presence of explicit time pressure as in the current study^31^. Failing to account for these collapsing bounds not only results in poorer model fits, it can also fundamentally alter one’s conclusions about other parameter estimates. Specifically, we show that collapsing bounds can be mimicked by joint changes in drift rate and threshold. As a result, variables that like value difference in the present study induce increases in drift rate will also artifactually increase threshold. Covariance among these parameters can therefore also serve as one diagnostic for latent threshold dynamics. Third, we show that overall value-related variability in decision threshold within a DDM framework can mimic stimulus-driven evidence accumulation within other evidence accumulation frameworks (i.e., without positing additional top-down mechanism for threshold control; Figure 4C). While our simulations demonstrate this using an LCA, the form of mimicry we describe generalizes to any other generative choice model that naturally gives rise to overall value-related speeding effects, including other parallel accumulator models^17,32^ and attentionally weighted DDMs^19^.

Collectively, these analyses undermine the empirical foundation for Vassena and colleagues’ motivated control account. While it cannot be ruled out that future studies well lend support for this account, this empirical gap encourages further reconsideration of the *normative* foundations of this account. Most notably, the model’s proposed mechanism requires that information about value (e.g., value difference) is available and used to selectively lower the decision threshold (e.g., when that difference is small). From first principles, there are problems with this requirement. First, the values of the options are precisely what the decision-maker seeks to identify as they make their decision. To the extent the decision-maker has access to these values directly, the decision process becomes obsolete. This concern indeed extends to our alternative DDM models. For similar reasons, existing models that account for online control adjustments do so not by relying on values per se, but instead on metacognitive representations that can be read out from the decision process (e.g., uncertainty or conflict^11,12,34,35^ or from longer-run statistics of the environment (e.g., reward rate^6,36^). Second, Vassena et al’s model predicts that, all else being equal, people will choose more quickly when choices are difficult relative to when they are easy – virtually all empirical research, including the author’s own data finds the exact opposite to be the case, both in the case of perceptual and value-based decision-making (e.g.,^37–39^). Third, the model draws inspiration (and intuitive support) from applications of adaptive control to decision-making in other cognitive tasks (e.g., flanker, Stroop). However, in these tasks control can be applied to enhance the signal associated with a predetermined stimulus, location, or feature based on an explicit response rule that defines relevant and irrelevant inputs. By contrast, the response to a value-based decision – and hence which information is relevant – is inextricably tied to the information being collected online, thus making the target of control at best a moving target. The leverage that any control mechanism has for enhancing evidence or speeding up the decision process is therefore limited.

While our current findings focus on the computational mechanisms underpinning choice behavior, they nevertheless carry significant implications for understanding neural mechanisms driving such decisions. For instance, one of the primary aims of Vassena and colleagues’ study was to test predictions of their previously proposed Predicted Response-Outcome model of dorsal anterior cingulate cortex (dACC)^15^. To provide a strong alternative to this model, they drew on a recent theory that proposes that dACC determines adjustments of cognitive control based on their overall expected value (EVC)^8,40^. The authors attributed to EVC the novel motivated control mechanism we critically re-examined here: response invigoration based on information about the values of options (Note that the original EVC model does not make this claim^21^). The authors supported the validity of this alternate model using their observation of value-based changes in response threshold, and proceeded to generate predictions about patterns of neural activity that should arise during the task. They then argued that activity in dACC during their task (greatest for the choices with the lowest value difference and the highest value difference) was inconsistent with predictions that they derived from this motivated control account, and by extension with EVC (see also ^21^ for additional concerns about how neural predictions were extrapolated from the EVC theory).

Our current findings raise a fundamental concern with this conclusion: their proposed motivated control account is unsupported by the findings from their experiment. Their critical finding that people selectively invigorate low value difference choices vanishes (and numerically reverses) when accounting for general choice dynamics in their task. This renders moot any neural predictions that would theoretically follow from such an adaptive control account, and may explain why Vassena and colleagues were unable to find evidence that bore out these neural predictions. It remains to be determined whether previous adaptive control accounts can quantitatively account for patterns of dACC activity observed in this study, but at a qualitative level those patterns can be accounted for by any theory that implicates the dACC in monitoring for potential conflict (low value differences) and surprise (high value differences), including EVC^1,8,21,40,41^.

Substantial progress has been made toward uncovering the neural and computational mechanisms at the intersection of value-based decision-making and cognitive control. This research flourishes when bold new mechanistic accounts are proposed and tested. Recent examples of this include work that normatively accounts for and empirically demonstrates dynamic adjustments in attention and evidence accumulation based on uncertainty and expected information gain^34,35,42^, and dynamic adjustments in threshold based on conflict^2,11,12^ or statistics of one’s choice environment ^6,36^. By providing a careful reexamination of both the empirical and normative basis of this recent and influential addition to these novel accounts, we hope that we have both raised the threshold for adopting such an account, while at the same time accelerating progress towards more fruitful accounts of when, whether, and how we control our decisions.

## Method

### Task Design.

Briefly, each trial presented two fractal images on the left and right of the screen (four total). Participants had to choose between the left pair and right pair within a 750 ms deadline, or they would miss out on their choice for that trial. Each fractal was worth between €0.10 and €0.80 (in increments of €0.10), and participants received the value of one fractal from the pair they chose, chosen randomly, in addition to their participant payment. Participants performed 160 trials, which were balanced with respect to the total value and difference between the left/right options. Additional details on this task are available in the original manuscript (Vassena et al., 2020).

### Participants.

Twenty-three participants (13 females; M_age_ = 23) were recruited, in accordance with the local research ethics committee. Twenty-two participants actually took part in the experiment.

### Drift Diffusion Analysis.

We fit drift diffusion models (DDMs) that both matched the authors original claims, and models that explored alternative explanations for their results. All models were fit using HDDM ^14,43^ , using five chains of 6000 samples (burning the first 2000 samples from each repetition). Model convergence was verified using the Gelman-Rubin R-hat statistic ^44^.

Apart from the Original model, models used the Likelihood approximation network (LAN) option in HDDM, providing efficient approximate likelihoods for collapsing bound DDMs. The Original model used the ‘HDDM’ function to predict correct responses, and the rest of the models used the ‘HDDMnnRegressor’ function to predict left/right choices. All models were fit with parameters at both the subject and group levels. As a requirement of the LAN fitting procedure, we used uninformed priors.

Models were compared using divergence information criteria (DIC;^45^). Note that the total number of trials differs between the Original and Original* models, as the Original model removed the most difficult choices. Normalizing the deviance by the sample size (‘trial-averaged likelihood’) still resulted in a DIC difference of 11.1 in favor of the Original* model, with the Original* model also offering a more comprehensive account of participants’ behavior. Parameter posteriors and posterior predictive checks were plotted using (Gaussian) kernel density estimation in MATLAB.

We validated our model-fitting and model-selection processes using model recovery (Supplementary Figure 3). We generated forty synthetic datasets from three models (Original*, VD static bound, VD collapsing bound) by sampling from the estimated parameter distributions for each participant. We then fit these three models to each generated dataset. To better match across fitting procedures, we fit all three models using HDDM’s likelihood approximation networks (though we found qualitatively similar results using the same fitting procedure as in the main experiment). We found that our model comparison procedure was biased towards collapsing bound models, with better DIC even when the data were generated by a static bound model. However, these biases were an order of magnitude smaller than the empirical differences in DIC that we found when we fit these models to participants. Given how unlikely these empirical differences were under the null model generated by model recovery, model recovery supports the conclusions in the main text.

We also validated that our estimated parameters were identifiable using parameter recovery. We generated forty synthetic datasets by sampling from the estimated parameter distributions for each participant (Supplementary Figure 5). Fitting the best-fitting modal to this synthetic dataset, we found that there was a good correspondence between the generating and estimated parameters, supporting their identifiability, and our ability to interpret the parameters.

**Table:** 

Model Name	Model Type	Regression Equation	Additional parameters
Original	ddm	v ~ [hard, easy] a ~ [hard, easy] z ~ [hard, easy]	z
Original*	ddm	v ~ −1 + signVD, a ~ 1 + absVD	z, p_outlier
VD	ddm	v ~ −1 + maxVD + minVD, a ~ 1 + absMaxVD + absMinVD	z, p_outlier
OV	ddm	v ~ −1 + maxVD + minVD, a ~ 1 + maxOV + minOV	z, p_outlier
VDOV	ddm	v ~ −1 + maxVD + minVD, a ~ 1 + absMaxVD + absMinVD + maxOV + minOV	z, p_outlier
VD both	angle	v ~ −1 + maxVD + minVD, a ~ 1 + absMaxVD + absMinVD, theta ~ 1 + absMaxVD + absMinVD	z, p_outlier, theta
VD init	angle	v ~ −1 + maxVD + minVD, a ~ 1 + absMaxVD + absMinVD	z, p_outlier, theta
VD rate	angle	v ~ −1 + maxVD + minVD, theta ~ 1 + absMaxVD + absMinVD	z, p_outlier, theta
OV both	angle	v ~ −1 + maxVD + minVD, a ~ 1 + maxOV + minOV, theta ~ 1 + maxOV + minOV	z, p_outlier, theta
OV init	angle	v ~ −1 + maxVD + minVD, a ~ 1 + maxOV + minOV	z, p_outlier, theta
OV rate	angle	v ~ −1 + maxVD + minVD, theta ~ 1 + maxOV + minOV	z, p_outlier, theta
VDOV both	angle	v ~ −1 + maxVD + minVD, a ~ 1 + absMaxVD + absMinVD + maxOV + minOV, theta ~ 1 + absMaxVD + absMinVD + maxOV + minOV	z, p_outlier, theta
VDOV init	angle	v ~ −1 + maxVD + minVD, a ~ 1 + absMaxVD + absMinVD + maxOV + minOV	z, p_outlier, theta
VDOV rate	angle	v ~ −1 + maxVD + minVD, theta ~ 1 + absMaxVD + absMinVD + maxOV + minOV	z, p_outlier, theta

*Model types:* ddm = static bound, angle = linear collapsing bound.

*Regression Equation:* v = drift rate, a = initial threshold, theta = collapse rate. signVD = signed value difference (equal weight), absVD = absolute value difference (equal weight). max/min VD = value difference for the maximum or minimum value options (e.g. max reflects the highest value left option and highest value right option). max/min OV = summed value for the max or min value options.

*Additional parameters:* z = starting point, p_outlier = lapse rate, theta = collapse rate.

### LCA simulations.

To obtain choices and response times we simulated 1000 iterations of each choice in the dataset using a custom LCA implemented in MATLAB. To account for the overweighting of the maximum values observed in the statistical analyses (cite), we set the inputs (I) to the accumulators set to the weighted average of the two values for each option (max value weight = ⅘, min value weight = ⅕). To account for the response deadline, we implemented a linearly collapsing bound (zb) that reaches zero at the deadline. We included a standard non-decision time, t0, which was set to 250ms. The initial bound height (z) was set to 10, the noise parameter (s) to 4.5, decay (k) to 0.153 and mutual inhibition (w) to 0.5. Accumulation was not bounded at zero, and activation could take negative values. Noise was normally distributed. Traces were simulated with a step size of 0.005. These parameters were hand-tuned to roughly approximate participants’ average accuracy and reaction time to generate realistic behavioral predictions.

The accumulator activation (AA) was updates at each step as:

$$\text{y}1_{\text{t+1}}=\text{y}1_{\text{t}}+\text{ste}{\text{p}}^{*}\left(\text{I}-{\text{k}}^{\text{*}}\text{y}1_{\text{t}}-{\text{w}}^{\text{*}}\text{y}2_{\text{t}}\right)+\mathrm{sqrt}{\left(\mathrm{step}\right)}^{*}\text{s(N})$$

$$\text{y}2_{\text{t+1}}=\text{y}2_{\text{t}}+\text{ste}{\text{p}}^{*}\left(\text{l}-{\text{k}}^{\text{*}}\text{y}1_{\text{t}}-{\text{w}}^{\text{*}}\text{y}1_{\text{t}}\right)+\mathrm{sqrt}{\left(\mathrm{step}\right)}^{*}\text{s(N})$$

Response times were registered as the timestep when one of the accumulators met or exceeded the current bound height, and the relevant accumulator was registered as the current choice. We used a random subset of these LCA simulations to fit the ‘Original*’ and ‘VDOV both’ HDDM models described above.

### Code Availability.

Data is available from the original authors, all code is available at xxxx.

## Supplementary Material

Supplement 1
