# Supplementary material for: Phantom controllers: Misspecified models create the false appearance of adaptive control during value-based choice
Source: bioRxiv. 2023 Jan 20:2023.01.18.524640. Preprint. [Version 1] doi: 10.1101/2023.01.18.524640 (PMC9882254; doi:10.1101/2023.01.18.524640)
Supplement: Supplementary file 1 [file NIHPP2023.01.18.524640v1-supplement-1.pdf]

bioRxiv preprint doi: https://doi.org/10.1101/2023.01.18.524640; this version posted January 20, 2023. The copyright holder for this preprint (which was not certified by peer review) is the author/funder, who has granted bioRxiv a license to display the preprint in perpetuity. It is made available under aCC-BY 4.0 International license.

Supplementary Figures

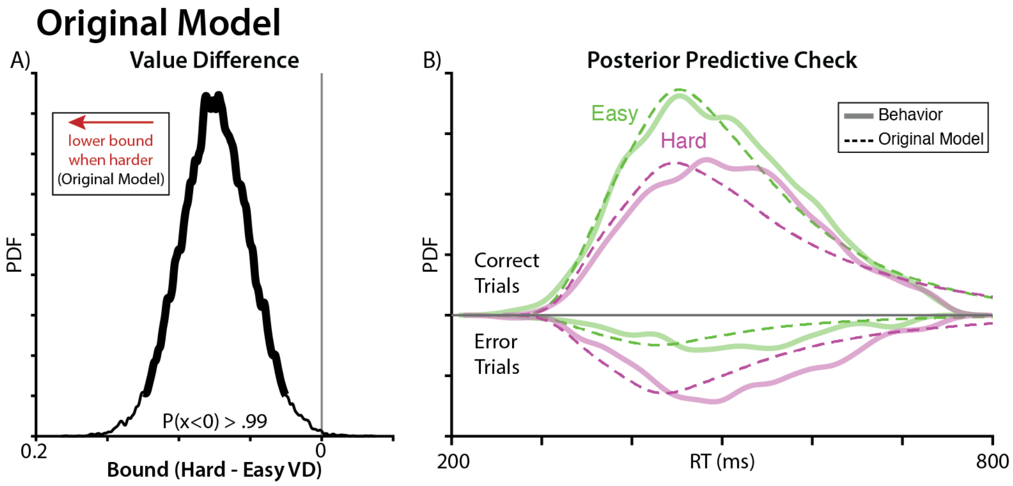

Supplementary Figure 1. Direct replication of analyses and findings in original paper. A) When fitting a model with the exact specifications used by Vassena et al., we replicate their finding of harder choices being associated with lower decision thresholds. **B)** Predicted choice behavior from this original model provides a poor qualitative fit to observed accuracy and reaction time distributions. See Figure 1C-D for a version of this model aimed at improving fit without altering the model's core assumptions.

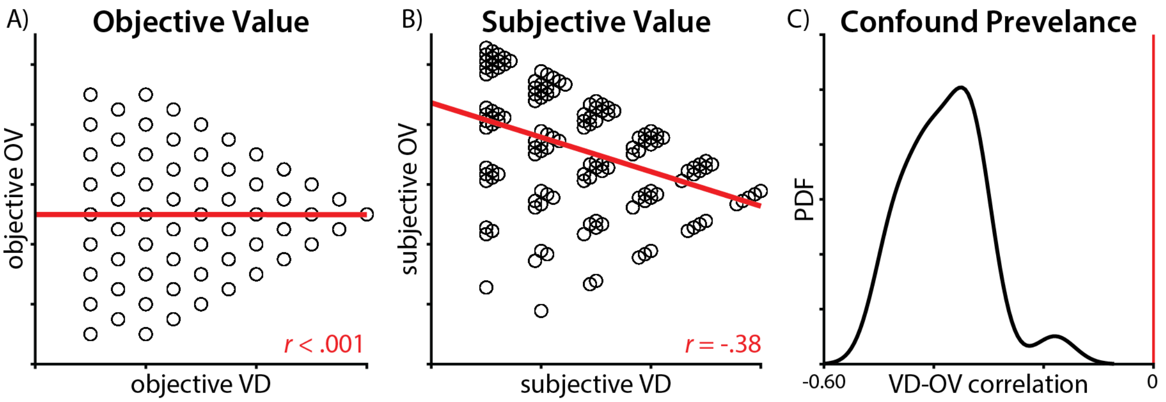

Supplementary Figure 2. Unequal weighting introduces confounding between overall value and value difference. A) If all outcomes were weighted equally (as in the Original and Original* models), value difference and overall value were uncorrelated by design. **B)** If participants have unequal weighting on the best and worst outcomes in each option (which improves model fit), value difference and overall value can become correlated. Here, we used the group-level drift rate weights estimated in the best-fitting model. **C)** Using the participant-specific drift rate weights, we found that all participants show some degree of correlation between overall value and value difference, introducing the potential for confounding.
